# Supplementary material for: Resolving the full spectrum of human genome variation using Linked-Reads
Source: Genome Res. 2019 Apr;29(4):635–45. doi: 10.1101/gr.234443.118 (PMC6442396; doi:10.1101/gr.234443.118)
Supplement: Supplemental Material [file supp_gr.234443.118_Supplemental_longranger-2.2.zip › longranger-2.2.tar.bz2!/lr2.2-public/lib/stan/cmdstan/stan/doc/api/html/get__lp_8hpp_source.html]

Stan: src/stan/math/matrix/get\_lp.hpp Source File


|  |  |
| --- | --- |
|  | Stan  2.5.0  probability, sampling & optimization |


- Main Page
- Related Pages
- Namespaces
- Classes
- Files

- File List
- File Members

All Classes Namespaces Files Functions Variables Typedefs Enumerations Enumerator Friends Macros Pages

- src
- stan
- math
- matrix

get\_lp.hpp

Go to the documentation of this file.

1 #include <boost/math/tools/promotion.hpp>

2 #include <stan/math/matrix/accumulator.hpp>

3

4 namespace stan {

5

6  namespace math {

7

8  template <typename T\_lp, typename T\_lp\_accum>

9  inline

10  typename boost::math::tools::promote\_args<T\_lp, T\_lp\_accum>::type

11  get\_lp(const T\_lp& lp,

12  const stan::math::accumulator<T\_lp\_accum>& lp\_accum) {

13  return lp + lp\_accum.sum();

14  }

15

16  }

17

18 }

stan::math::accumulator::sum

T sum() const

Return the sum of the accumulated values.

**Definition:** accumulator.hpp:113

accumulator.hpp

stan::math::get\_lp

boost::math::tools::promote\_args< T\_lp, T\_lp\_accum >::type get\_lp(const T\_lp &lp, const stan::math::accumulator< T\_lp\_accum > &lp\_accum)

**Definition:** get\_lp.hpp:11

stan::math::accumulator

Class to accumulate values and eventually return their sum.

**Definition:** accumulator.hpp:25


---

[ Stan Home Page ]

*© 2011–2014,
Stan Development Team.*
